# Vaccine‐Associated Recombination and Virulence Heterogeneity of GI‐19 (QX Type) Infectious Bronchitis Virus in Eastern and Southern China, 2024−2025

**DOI:** 10.1155/tbed/8100600

**Published:** 2026-02-23

**Authors:** Zijian Dai, Yuanlu Lu, Lulu Deng, Yan Luo, Yiran Zeng, Yusen Tian, Xianchen Meng, Haitao Zhang, Jihui Ping

**Affiliations:** ^1^ MOE Joint International Research Laboratory of Animal Health and Food Safety, Engineering Laboratory of Animal Immunity of Jiangsu Province, College of Veterinary Medicine, Nanjing Agricultural University, Nanjing, China, njau.edu.cn; ^2^ Shangqiu Polytechnic, Shangqiu, China; ^3^ Lihua Nanjing Industrial Research Institute Co., Ltd., Nanjing, China

**Keywords:** antigenic variation, genome recombination, infectious bronchitis virus, molecular epidemiology, virulence heterogeneity

## Abstract

Infectious bronchitis (IB) virus (IBV) remains a major pathogen threatening the poultry industry. Its rapid mutation and recombination continuously generate variants that disseminate worldwide. Between May 2024 and February 2025, 49 field strains were isolated from chickens vaccinated with live‐attenuated IBV vaccines (H120, 4/91, or QXL87) in four Chinese provinces (Jiangsu, Anhui, Shandong, and Guangdong). Based on full‐length S1 gene sequencing, all isolates were classified into genotype GI, including lineages GI‐13 and GI‐19. Phylogenetic analysis revealed that GI‐19 (QX‐type) comprised as much as 83.67%, with the nucleotide homology of the S1 gene to QXL87 varying from 93.4% to 99.8%. Recombination analysis indicated that the S1 genes of three isolates incorporate QXL87 and 4/91 genetic material, possibly arising from recombination between the QX‐type and 4/91 vaccine strains. Virulence assessment in 1‐day‐old specific‐pathogen‐free (SPF) chickens demonstrated that four phylogenetically distant QX‐type strains and one recombinant strain (with QXL87 as the major parent) induced varying degrees of tissue damage and mortality. Cross‐neutralization assays demonstrated reduced antigenic relatedness between the circulating isolates and QXL87 vaccine strain. Structural mapping analysis further indicated that three amino acid mutations within the N‐terminal domain (NTD) and two amino acid mutations in the C‐terminal domain (CTD) of the S1 subunit alter its overall conformation, potentially leading to antigenic variation and facilitating immune evasion. Overall, these findings offer timely insights into the epidemiology and virulence heterogeneity of QX‐IBV, providing valuable references for optimizing vaccine selection and development, as well as for preventing and controlling the disease.

## 1. Introduction

Infectious bronchitis (IB) is an acute and highly contagious viral disease in chickens caused by the IB virus (IBV) [1]. The first documented IB outbreak occurred in North Dakota, United States, in 1930, and the disease was subsequently introduced to China in the 1950s [2]. IBV is classified within the *Gammacoronavirus* genus and possesses a non‐segmented, single‐stranded, and positive‐sense RNA genome ~27.6 kb in length. The virions are roughly 80–120 nm in diameter, enveloped, and feature distinctive club‐shaped surface projections [3]. The structural proteins of the virus include the spike (S), envelope (E), membrane (M), and nucleocapsid (N) proteins. The S protein undergoes proteolytic cleavage inside the host cell, resulting in an N‐terminal S1 subunit and a C‐terminal S2 subunit. The S1 subunit contains the primary receptor‐binding domain (RBD) responsible for cellular attachment and constitutes the major protective antigen, whereas the S2 subunit facilitates fusion between the viral envelope and the host cell membranes [4].

In China, IBV currently circulates as a complex mixture of multiple genotypes. Based on phylogenetic analyzes of the S1 gene, nine genotypes (GI–GIX) have been delineated. The GI genotype alone comprises 42 distinct lineages [5–7]. Among these, the QX‐type (GI‐19) predominates throughout China. Highly pathogenic QX‐type strains exhibit broad tissue tropism, causing severe respiratory symptoms (coughing, tracheal rales) and pronounced nephropathology characterized by visceral urate deposition; case fatality rates can reach 60% [8]. A second widely disseminated genotype, 4/91‐type (GI‐13), first emerged in Europe in 1991 and subsequently spread worldwide [9]. Notably, 4/91‐type strains display extensive recombinogenic potential and readily exchange genetic fragments with the QX‐type strains, further complicating the epidemiological landscape [10].

Currently, IB is primarily controlled by vaccination with live‐attenuated or inactivated vaccines. However, the high mutation rate of IBV and its propensity for inter‐strain recombination significantly reduce cross‐protective efficacy among antigenically distinct serotype or genotype strains. Consequently, current vaccines fail to confer robust immunity against circulating field strains, facilitating viral escape and complicating disease control [11, 12]. In China, despite routine immunization using vaccine strains corresponding to the major genotypes (H120, 4/91, or QXL87), outbreaks continue to occur in vaccinated flocks. Field isolates are dominated by wild‐type QX (GI‐19) and 4/91 (GI‐13) lineages, and vaccine pressure appears to have accelerated the emergence of recombinant variants [8, 13].

To refine our understanding of IBV molecular epidemiology in eastern and southern China, we conducted a surveillance program between May 2024 and February 2025. A total of 49 IBV strains were isolated from commercial yellow chicken flocks across four provinces (Shandong, Jiangsu, Anhui, and Guangdong), all of which had been vaccinated with H120, 4/91, or QXL87 vaccines. Phylogenetic analysis of the complete S1 gene revealed that all isolates clustered within either GI‐19 (QX‐type) or GI‐13 (4/91‐type) lineage. Notably, QX‐type isolates exhibited significant genetic divergence from the QXL87 vaccine strain (S1 amino acid identity 93.4%−99.8%), forming several predominant clades represented by four strains: CK/CH/XZ/240457 (XZ240457), CK/CH/CZ/240601 (CZ240601), CK/CH/WF/24054 (WF24054), and CK/CH/WF/526 (WF526). This antigenic drift may be a key factor in the suboptimal protection conferred by current vaccines. Recombination analysis targeting the S1 region identified three isolates originating from the QX‐type and 4/91‐type parental strains. Virulence testing in specific‐pathogen‐free (SPF) chickens with four representative QX‐type isolates and the recombinant strain AH240519 (with QXL87 as the major parental strain) resulted in variable mortality rates and characteristic histopathological lesions in respiratory and kidney tissues. Collectively, these findings offer valuable insights into the evolution of IBV and provide essential evidence for refining vaccination strategies tailored to regional needs.

## 2. Materials and Methods

### 2.1. Eggs and Chickens

SPF chicken embryos and 1‐day‐old SPF chickens were supplied by Jinan SAIS Poultry Co. Ltd., China. The chickens were housed in isolation units with negative pressure throughout the experiment, and water and feed were provided ad libitum.

### 2.2. Sample Collection and Virus Isolation

Between May 2024 and February 2025, a total of 540 oropharynx and cloacal swabs were collected from sick and weak chickens exhibiting respiratory symptoms, including coughing, wheezing, rales, and nasal discharge. The samples were collected from chicken flocks across four provinces in China: Shandong, Jiangsu, Anhui, and Guangdong. All flocks had been vaccinated with live‐attenuated H120 (FJ888351), 4/91 (KF377577), or QXL87 (MH743141) vaccines supplied by QYH Biotech Co., Ltd. (Nanjing, China). To isolate the virus, the samples were freeze‐thawed three times at −80°C and then centrifuged at 5000 rpm for 10 min to obtain the supernatant. The supernatant was propagated through three blind passages in 9‐day‐old SPF chicken embryos via the allantoic cavity. Allantoic fluid was collected for RNA extraction using TRIzol Reagent (Accurate Biology, Changsha, China) and subsequently analyzed by real‐time fluorescence quantitative polymerase chain reaction (RT‐qPCR) using AceQ qPCR SYBR Green Master Mix (Q111‐02, Vazyme, China). The primers used for IBV identification were described in the previous study [14]. The reaction conditions were as follows: 50°C for 15 min, 95°C for 30 s, and 45 cycles of 95°C for 10 s and 60°C for 30 s, performed on an Applied Biosystems 7500 system (Thermo Fisher, Beijing, China). A cycle threshold (CT) value <30 per 2 µL RNA template was used as the criterion for positivity. In addition, embryos were examined for characteristic lesions to further confirm viral detection.

### 2.3. S1 Gene Phylogenetic Analysis and Prevalence Analysis

The S1 gene of IBV was amplified by reverse transcription PCR (RT‐PCR) using the HiScript II One Step RT‐PCR Kit (P612‐01, Vazyme, China), and the primers are listed in Table S1. The viral RNA was prepared as described previously. The PCR parameters were as follows: 95°C for 10 min, 30 cycles of 95°C for 15 s, 58°C for 15 s, and 72°C for 1.5 min, followed by a final step of 72°C for 10 min. The amplified products were separated on an agarose gel and sequenced by General Biol Corporation (Anhui, China). The full‐length S1 amino acid sequences of the isolates (including 540 amino acids), along with reference sequences from nine genotypes (GI‐GIX) and some recently reported novel lineages [11, 15], were aligned using MEGA (version 11.0) [16]. Reference sequences were selected from GenBank based on their clear genotype annotations and relevance to the strains analyzed in this study, ensuring representative coverage for phylogenetic analysis [5] (Table S2). Phylogenetic analysis of the S1 gene sequences was performed with 1000 bootstrap replicates using the neighbor‐joining method. The phylogenetic tree was visualized using the Interactive Tree of Life (iTOL) web‐based tool [17].

### 2.4. S1 Gene Recombination Analysis

Recombination analysis was performed based on the S1 gene of IBV isolates using the Recombination Detection Program 4 (RDP v.4.97) software [18]. A total of seven methods implemented in RDP4 were applied, including RDP, GENECONV, 3Seq, Chimaera, SiScan, MaxChi, and LARD. Recombination detected by at least three of the seven methods with a *p* ≤ 10^−12^ was considered true recombination [19]. Potential recombination events were further verified by similarity plot analysis using SimPlot version 3.5.1 [20].

### 2.5. Viral Growth Kinetics Analysis

To evaluate the replication ability of isolates in vitro, 100 EID_50_ of IBV isolates were inoculated into 9‐day‐old SPF embryos. At 12, 24, 36, 48, 60, and 72 h postinfection (hpi), 500 μL of allantoic fluid was collected from each embryo for RNA extraction and viral load was quantified by RT‐qPCR. As described previously [21], RT‐qPCR was performed using primers F_391_ (5′‐GCTTTTGAGCCTAGCGTT‐3′) and R_533_ (5′‐GCCATGTTGTCACTGTCTATTG‐3′), and the standard curve equation was *y* = −3.315*x* + 39.704 (*R* ^2^ = 0.9991). All experiments were performed in triplicate, and the viral copy numbers were calculated based on the standard curve.

### 2.6. Isolates Selected for Virulence Test

To evaluate the virulence of the QX‐type isolates, five strains (including four GI‐19 strains and one recombinant strain, AH240519, with QXL87 as the major parent) that exhibited over 5% S1 amino acid divergence from the QXL87 vaccine strain and belonged to distinct major phylogenetic clades were selected. In this study, 120 1‐day‐old SPF chickens were randomly allocated into six groups of 20. Chickens in the infected group were infected with 10^5.0^ EID_50_ of AH240519, XZ240457, CZ240601, WF24054, or WF526 via ocular and intranasal routes (0.2 mL per chicken), while the control group received the same dose of PBS. The infection day was designated as 0 days postinfection (dpi). Clinical signs and mortality were monitored and recorded daily for 14 days. At 3, 6, 9, and 14 dpi, two chickens from each group were randomly selected for euthanasia and autopsy. Tissue samples from the trachea, lungs, and kidneys were collected for histopathological examination and viral load quantification. Additionally, oropharynx and cloacal swabs were collected from four to six chickens per group at each time point to evaluate viral shedding.

### 2.7. Histopathological Examination

The trachea, lung, and kidney tissues, collected at 6 dpi, were fixed in 10% neutral formalin for 48 h at room temperature. Subsequently, the samples were routinely processed, embedded in paraffin wax, cut into 5 µm thin sections, and stained with hematoxylin and eosin (H&E) for observation under light microscopy (TS100, Nikon, Japan).

### 2.8. Viral Load Determination in Tissues

Total RNA was extracted from tissues and swabs using TRIzol Reagent. cDNA was synthesized from 1 μg of total RNA per sample with random primers and the HiScript II 1st Strand cDNA Synthesis Kit. Viral loads in tissues and virus shedding were quantified by RT‐qPCR. As previously mentioned, viral copy numbers were calculated based on the standard curve, and all experiments were conducted in triplicate.

### 2.9. Viral Cross‐Neutralization Assay and Serotype Analysis

Blood samples were collected from surviving chickens in each group at 21 dpi, and anti‐IBV sera were subsequently isolated. To evaluate the antigenic relatedness between the IBV isolates and the QXL87 vaccine strain, virus cross‐neutralization assays were performed using anti‐QXL87 serum provided by Lihua Nanjing Industrial Research Institute Co., Ltd. (Nanjing, China). All serum samples were inactivated at 56°C for 30 min and then serially diluted two‐fold with PBS. In parallel, the virus suspension was diluted to 200 EID_50_ with PBS. Equal volumes of diluted serum and virus were mixed and incubated at 37°C for 1 h. The virus‐serum mixtures were then inoculated into the allantoic cavity of 9‐day‐old SPF chicken embryos [22]. After 144 h, the embryonic lesions were observed and counted, and the neutralization titers of each group were calculated using the Reed and Muench [23] methods. The virus cross‐neutralization *R* value for each strain was calculated as previously described [24]. The criteria for antigenic relationships were defined according to a previously reported method for IBV [25]: cross‐reactivity *R* values ≥0.70 indicate antigenic identity; 0.70 >*R* ≥0.33 indicates antigenic relatedness (minor subtype differences); 0.33 >*R* ≥0.11 indicates distant relatedness (major subtype differences); whereas *R* <0.11 indicates no antigenic relatedness (serotype differences).

### 2.10. S1 Amino Acid Residue Mutation and Antigenic Variation Analysis

To identify S1 residues that determine IBV antigenicity, sequence alignments were performed between the five isolates and QXL87 and visualized with ESPript 3.0 [26]. Critical residues were mapped based on the full M41 spike cryo‐EM structure (PDB: 6CV0) [27, 28] and its monomeric S1 subunit. Homology models of the S1 domains were generated with SWISS‐MODEL (https://swissmodel.expasy.org/), using 6CV0 as the template [29], and superpositions were performed in PyMOL (v2.5.4) to assess the structural impact of variant amino acid residues.

### 2.11. Statistical Analysis

Data were analyzed using GraphPad Prism version 7.0 (GraphPad, La Jolla, CA, USA). Statistically significant differences were evaluated by performing a two‐way analysis of variance (ANOVA) ( ^∗^, *p* < 0.05;  ^∗∗^, *p* < 0.01;  ^∗∗∗^, *p* < 0.001;  ^∗∗∗∗^, *p* < 0.0001).

## 3. Results

### 3.1. Prevalence and Phylogenetic Analysis of IBV

Between 2024 and 2025, 168 out of 540 samples tested positive by RT‐qPCR, resulting in a positivity rate of 31.11% (Table S3). A total of 49 IBV strains were successfully isolated from four Chinese provinces: Jiangsu (*n* = 12, 24.49%), Anhui (*n* = 22, 44.90%), Shandong (*n* = 13, 26.53%), and Guangdong (*n* = 2, 4.08%) (Figure 1A). The GenBank accession numbers for the isolates ranged from PX737632 to PX737680, and their background information was listed (Table S4). The S1 amino acid sequences were aligned in MEGA 11.0 against nine reference genotypes, and the resulting phylogeny was visualized using iTOL. All isolates were classified into the GI genotype, predominantly GI‐19 (*n* = 40, 81.63%), followed by GI‐13 (*n* = 6, 12.24%) and recombinants of GI‐19 and GI‐13 (*n* = 3, 6.12%) (Figure 1B).

Figure 1Prevalence and phylogenetic analysis of IBV based on the S1 gene. (A) Geographic distribution of IBV isolates in four Chinese provinces surveyed between May 2024 and February 2025. Provincial boundaries were generated with DataV GeoAtlas (https://datav.aliyun.com/portal). The color intensity reflects the number of IBV isolates detected in each province. (B) Phylogenetic tree of S1 nucleotide sequence. The tree was constructed in MEGA (version 11.0) using the neighbor‐joining method. The dataset included 81 reference sequences representing nine genotypes (GI‐GIX) and 49 field isolates. The GI‐19 and GI‐13 clades are highlighted in light red and light blue, respectively. Three recombinant strains are represented by yellow hexagons, while the four predominant QX‐type isolates (CK/CH/XZ/240457, CK/CH/CZ/240601, CK/CH/WF/24054, and CK/CH/WF/526) are marked with black stars. The vaccine strains 4/91 and QXL87 are shown as black triangles, and reference strains are indicated by black circles.

**Figure:**
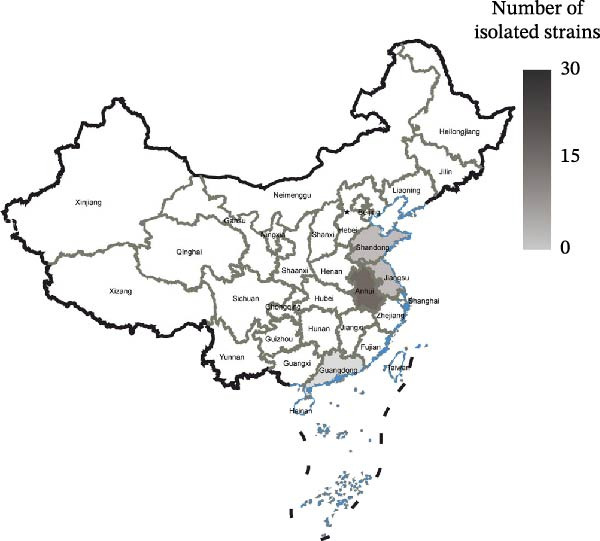
(A)

**Figure:**
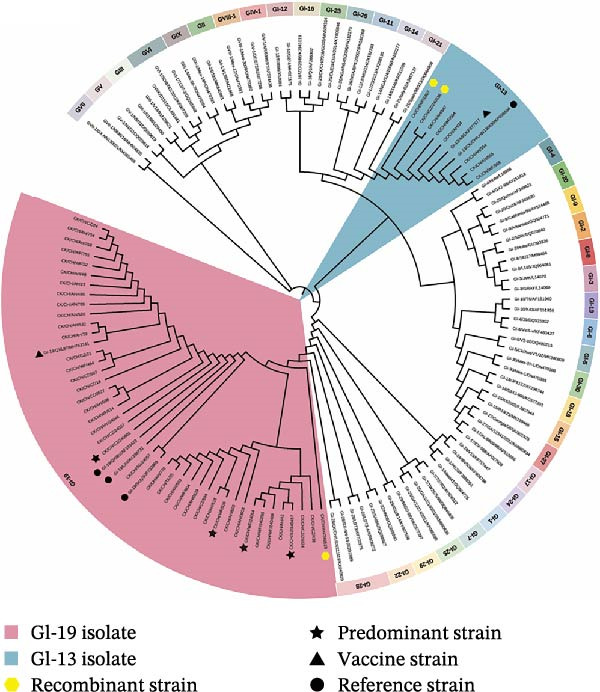
(B)

### 3.2. High Diversity of the S1 Gene

To further investigate IBV diversity, S1 amino acid sequences were compared, revealing significant divergence in pairwise identities. Nucleotide and amino acid similarities ranged from 77.6% to 100% and 76.9% to 100%, respectively. The greatest divergence (77.6% for nucleotide and 76.9% for amino acid sequences) was observed between the isolates CK/CH/AH/0529 and CK/CH/AH/555. Subsequently, the isolated sequences were aligned with the currently used live‐attenuated vaccine strains (QXL87, 4/91, or H120) in the field (Table 1). Consistent with the recurrent QX‐type outbreaks in vaccinated flocks in China, the GI‐19 isolates were more distantly related to QXL87 (S1 amino acid identity ranging from 93.4% to 99.8%), while the GI‐13 isolates exhibited minimal divergence from the 4/91 vaccine strain (with identities ranging from 98.9% to 99.1%). Consequently, the genetic divergence between the GI‐19 isolates and QXL87 was selected as the focus of subsequent analyzes. Phylogenetic reconstruction revealed that the five isolates, AH240519, XZ240457, CZ240601, WF24054, and WF526, formed independent evolutionary branches and evolved at a certain distance from QXL87. The S1 amino acid homology of the five isolates to QXL87 was 89.4% (AH240519), 93.9% (XZ240457), 95.0% (CZ240601), 93.3% (WF24054), and 93.9% (WF526), respectively.

**Table 1:** Similarity in nucleotides and amino acids between isolates and vaccine strains.

Isolated strains of various genotypes	Vaccine strains		
	QXL87	4/91	H120
GI‐19 (QX) (*n* = 36)	94.0%−99.8% (nt)/93.4%−99.8% (aa)	78.0%−78.4% (nt)/77.0%−78.3% (aa)	76.6%−77.1% (nt)/75.3%−76.6% (aa)
GI‐13 (4/91) (*n* = 6)	77.9%−78.0% (nt)/77.9%−78.5% (aa)	98.7%−99.3% (nt)/98.9%−99.1% (aa)	78.0%−78.2% (nt)/74.8%−75.1% (aa)
AH240519^a,b^ (*n* = 1)	89.3% (nt)/89.4% (aa)	86.3% (nt)/84.8% (aa)	78.2% (nt)/75.9% (aa)
WF2407^a^ (*n* = 1)	79.4% (nt)/81.4% (aa)	97.3% (nt)/97.0% (aa)	77.2% (nt)/74.6% (aa)
CZ240536^a^ (*n* = 1)	81.0% (nt)/82.6% (aa)	96.7% (nt)/95.7% (aa)	77.1% (nt)/74.0%(aa)
XZ240457^b^ (*n* = 1)	94.3% (nt)/93.9% (aa)	78.1% (nt)/77.8% (aa)	77.3% (nt)/75.9% (aa)
CZ240601^b^ (*n* = 1)	95.4% (nt)/95.0% (aa)	78.0% (nt)/78.0% (aa)	77.1% (nt)/75.4% (aa)
WF24054^b^ (*n* = 1)	94.3% (nt)/93.3% (aa)	78.0% (nt)/77.8% (aa)	77.2% (nt)/75.9% (aa)
WF526^b^ (*n* = 1)	94.4% (nt)/93.9% (aa)	78.4% (nt)/78.0% (aa)	77.3% (nt)/75.8% (aa)

^a^Three recombinant strains derived from the recombination between the QX and 4/91 strains in the S1 gene region.

^b^Five IBV strains used for the virulence experiment in chickens.

### 3.3. Triple Recombinants of 4/91 and QX Type

Recombination events within the S1 gene were analyzed using RDP4 software. Events supported by at least three of the seven methods (*p* ≤ 1 × 10^−12^) were considered genuine. The complete S1 genes of the 49 IBV isolates and reference strains were aligned to identify the recombinants. Among these, the recombination events were detected in the S1 gene of three isolates (Table S5). AH240519 resulted from recombination between 4/91 and QXL87, with QXL87 contributing the larger segment (major parent) from the 5′ end to nucleotide 986, while 4/91 provided the smaller 3′ end fragment. In contrast, CZ240536 and WF2407 exhibited the reverse pattern: 4/91 provided the backbone (major parent), and QXL87 contributed discrete internal regions demarcated by breakpoints at positions 1365–1620 and 1–123, respectively. These recombination events significantly increased the genetic diversity of IBV and underscore the modular evolution of the S1 region. To further characterize these three recombination events, BootScan analysis using Simplot was performed with 4/91 and QXL87 as reference strains (Figure 2). Notably, 4/91 participated in recombination more frequently than any other vaccine strain examined, acting as either a donor or a recipient.

Figure 2SimPlot analysis of recombination within the complete S1 gene. Colored lines denote reference strains: red, QXL87 vaccine strain; blue, 4/91 vaccine strain. (A) Recombinant CK/CH/AH/240519 (AH240519), derived from QXL87 and 4/91, with a recombination breakpoint at 986 nt (indicated by a black arrow). (B) Recombinant CK/CH/CZ/240536 (CZ240536), derived from 4/91 and QXL87, with a breakpoint at 1365 nt (indicated by a black arrow). (C) Recombinant CK/CH/WF/2407 (WF2407), derived from 4/91 and QXL87, with a breakpoint at 123 nt (indicated by a black arrow).

**Figure:**
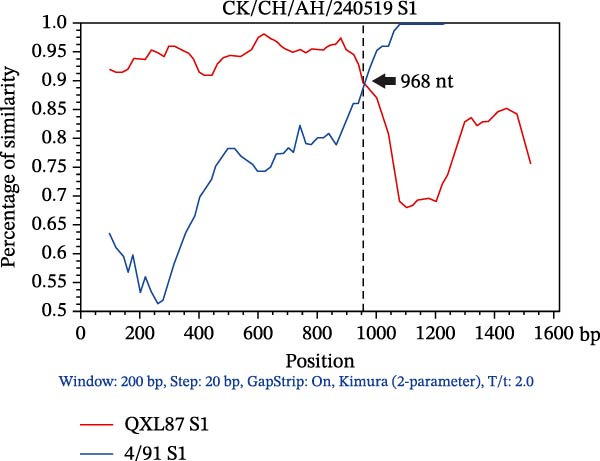
(A)

**Figure:**
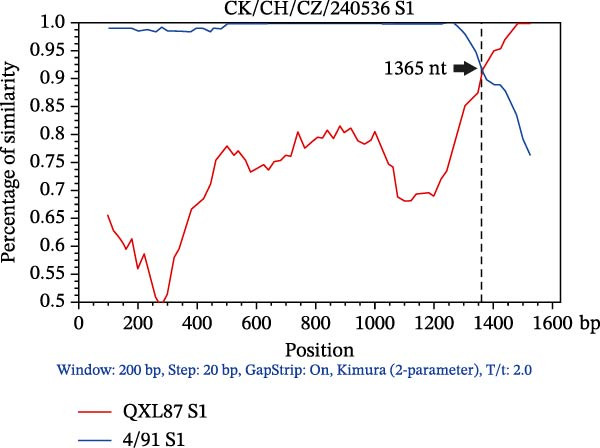
(B)

**Figure:**
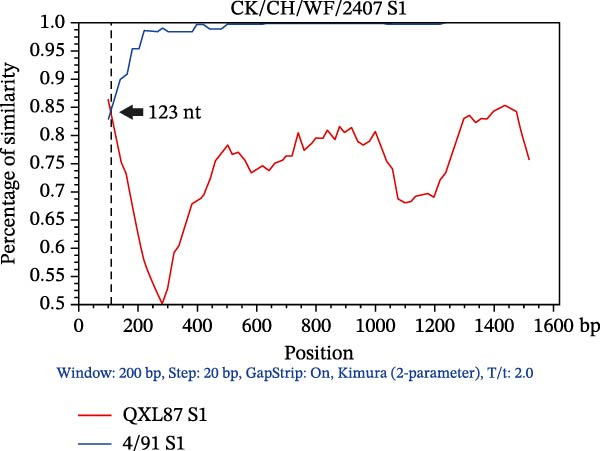
(C)

### 3.4. Biological Characteristics of Five QX‐Type Isolates

As previously described, four QX‐type isolates and the recombinant strain AH240519, each forming independent phylogenetic branches, were selected to investigate the biological characteristics of the ongoing QX‐type outbreaks. Each isolate was purified through three consecutive rounds of limiting dilution (Figure 3A), and the resulting clonal stocks were inoculated into the allantoic cavities of 9‐day‐old SPF embryos. After 6 dpi, all embryos exhibited severe stunting and generalized dwarfism (Figure 3B). To quantify viral infectivity, serial 10‐fold dilutions of clarified allantoic fluid in PBS were inoculated into 9‐day‐old SPF embryos. Specific lesions were recorded at 6 dpi, and the EID_50_ was calculated using the Reed and Muench method (Figure 3C). Growth kinetics were then compared by inoculating 100 EID_50_ of each isolate into SPF embryos and harvesting allantoic fluid at 12, 24, 36, 48, 60, and 72 hpi. Viral RNA copies were quantified by RT‐qPCR, and growth curves were constructed. AH240519, the recombinant strain incorporating 4/91‐derived sequences, displayed significantly higher titers at 24 hpi compared with the other four isolates (*p* < 0.01, *p* < 0.001, *p* < 0.001, and *p* < 0.001) (Figure 3D), suggesting that recombination with 4/91 may confer a short‐term replicative advantage. In contrast, WF526 exhibited the lowest viral load across all time points, suggesting attenuated virulence.

Figure 3Biological characterization of the five predominant IBV isolates. (A) Specificity screening. Viral RNA extracted from allantoic fluid was tested by RT‐PCR for IBV, avian influenza virus (AIV), Newcastle disease virus (NDV), and infectious bursal disease virus (IBDV). Lanes 1–5 correspond to isolates AH240519, XZ240457, CZ240601, WF24054, and WF526, respectively. (B) Embryo pathogenicity. Each isolate was inoculated into the allantoic cavity of 9‐day‐old SPF chicken embryos, and lesions (stunting or dwarfing) were recorded at 6 dpi. (C) Virus titration. Serial 10‐fold dilutions of virus‐containing allantoic fluid in PBS were inoculated into 9‐day‐old SPF embryos, and EID_50_ values were calculated after 6 dpi by the Reed and Muench method [23]. (D) Replication kinetics. After allantoic inoculation of 9‐day‐old SPF embryos, viral RNA loads were quantified by RT‐qPCR at 12, 24, 36, 48, 60, and 72 hpi.

**Figure:**
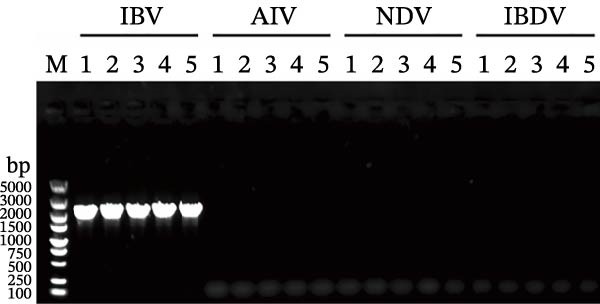
(A)

**Figure:**
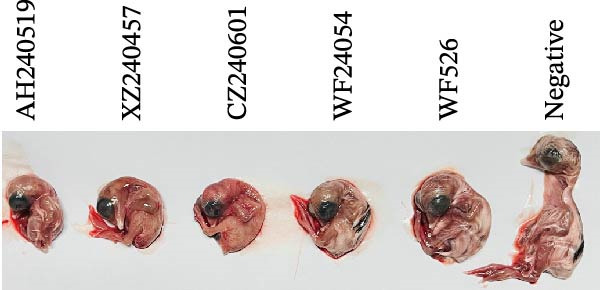
(B)

**Figure:**
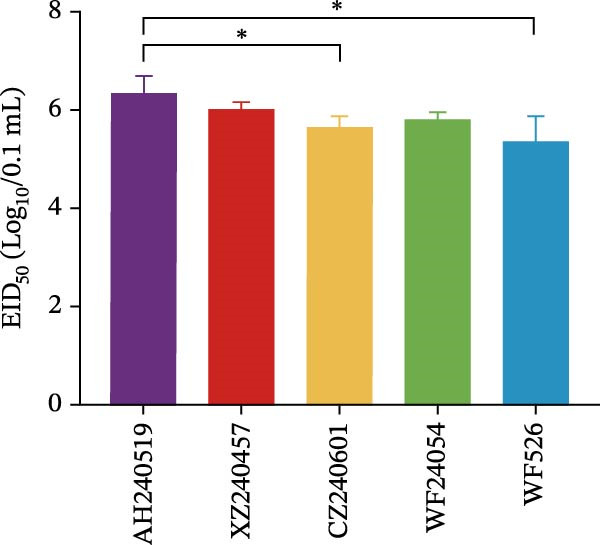
(C)

**Figure:**
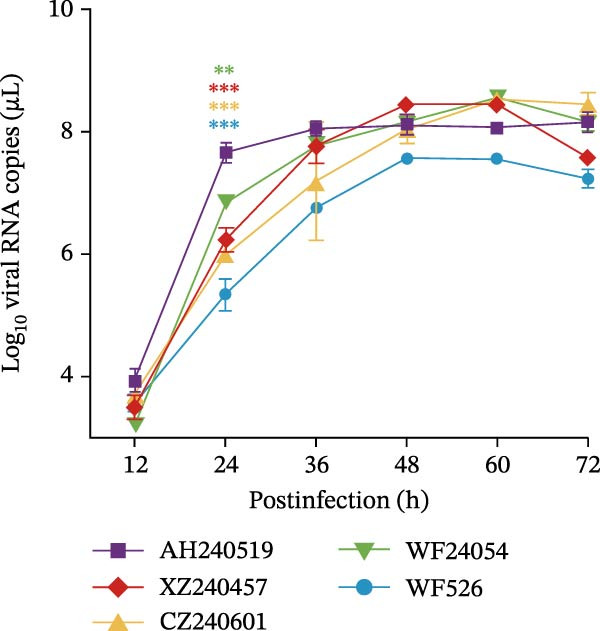
(D)

### 3.5. Virulence Assessment of QX‐Type Isolates in SPF Chickens

The virulence of the five isolates was evaluated in 1‐day‐old SPF chickens (*n* = 20 per group) inoculated with 10^5^ EID_50_ per chicken via the ocular and nasal routes, with a PBS‐inoculated group serving as the negative control. Clinical signs and mortality were monitored daily for 14 days (Figure 4A and B). The isolates exhibited distinct virulence profiles. Chickens challenged with AH240519 and XZ240457 developed severe respiratory distress and exhibited mortality rates of 40% and 50%, respectively. In contrast, infection with CZ240601 or WF24054 resulted in milder disease with a 20% mortality rate, whereas WF526 induced only transient, mild clinical signs and 100% survival, despite sharing the same GI‐19 backbone. Chickens inoculated with PBS remained clinically normal throughout the observation period. Oropharynx and cloacal viral shedding remained consistently high at 3, 6, 9, and 14 dpi in all challenged groups, except for WF526 at 3 dpi (*p* < 0.01 or *p* < 0.001) (Figure 4C). This sustained and robust viral excretion suggests that, apart from the attenuated WF526 strain, the remaining QX isolates possessed significantly enhanced transmissibility, posing a substantial threat to poultry health. RT‐qPCR analysis of tissue homogenates from the trachea, lungs, and kidneys revealed peak viral loads in these tissues at 6–9 dpi, followed by a gradual decline, although viral RNA remained detectable at 14 dpi. AH240519 and XZ240457 exhibited significantly higher viral genome copies than the other groups (*p* < 0.05 or *p* < 0.01) (Figure 4D–F), confirming pronounced tropism for both the respiratory and urinary tracts and sustained viral shedding corresponding to their higher virulence.

Figure 4Daily clinical scores, survival rates, viral shedding, and viral loads in different tissues. (A) Daily clinical scores. Scoring system: 0 = normal; 1 = mild nasal discharge, lacrimation, or head shaking; 2 = watery droppings, depression, coughing, or sneezing; 3 = severe nasal exudate, marked depression, open‐mouth breathing, or tracheal rales; 4 = death. (B) Survival curve. The percentage of surviving chickens in each group is presented over a 14‐day observation period. (C) Viral shedding. Viral RNA was extracted from oropharynx and cloacal swabs and quantified by RT‐qPCR. (D–F) Viral load quantification. Viral RNA levels in the trachea, lungs, and kidneys were measured by RT‐qPCR at the indicated time points.

**Figure:**
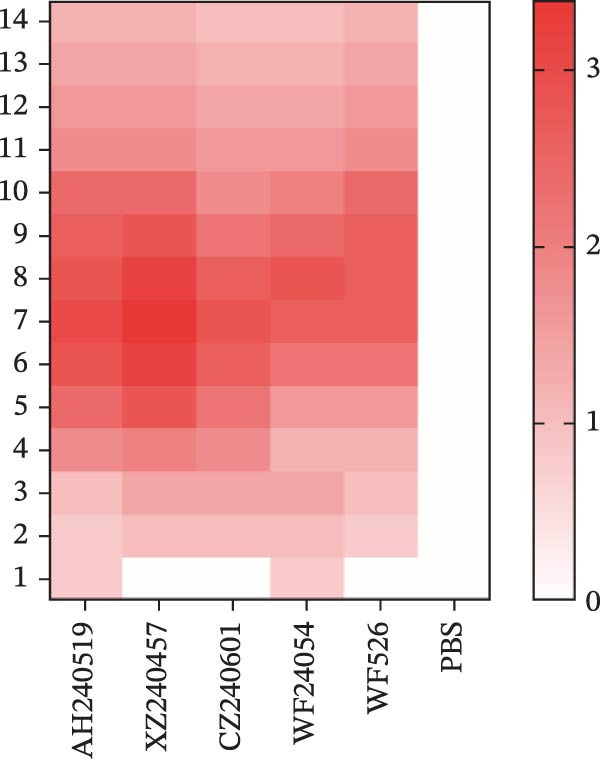
(A)

**Figure:**
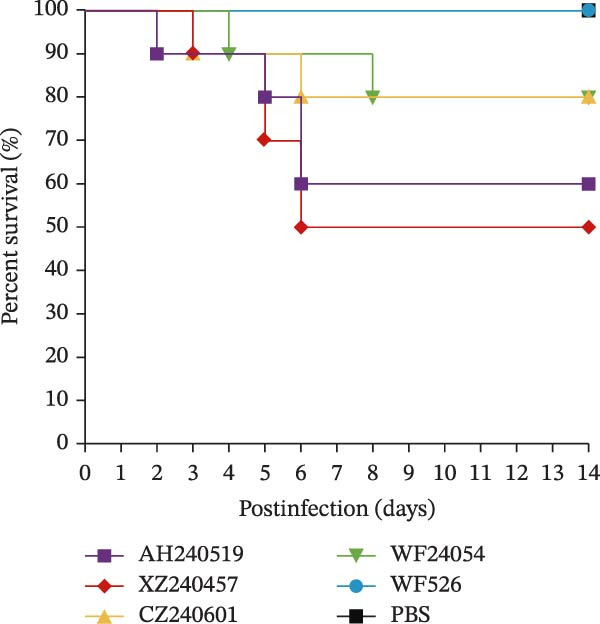
(B)

**Figure:**
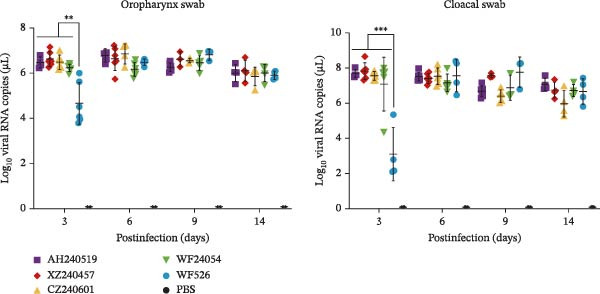
(C)

**Figure:**
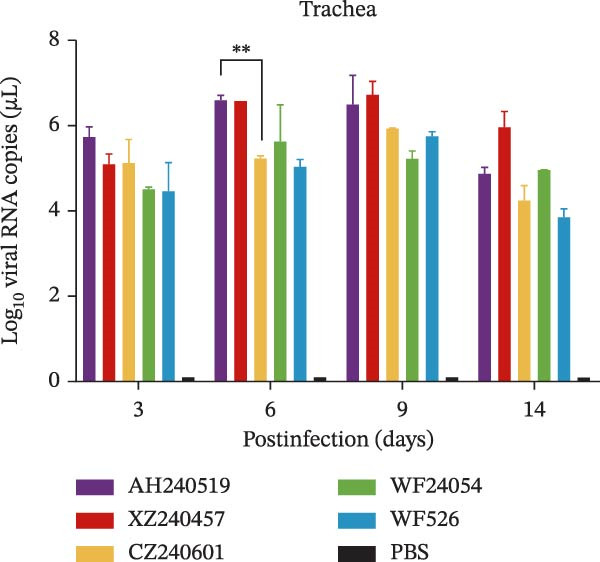
(D)

**Figure:**
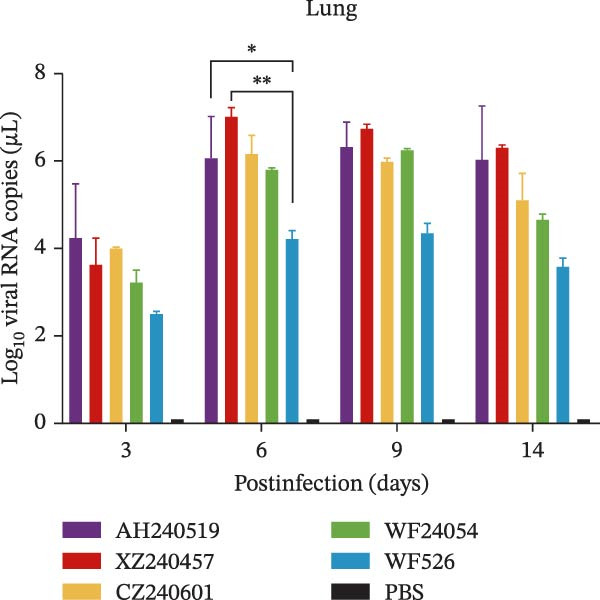
(E)

**Figure:**
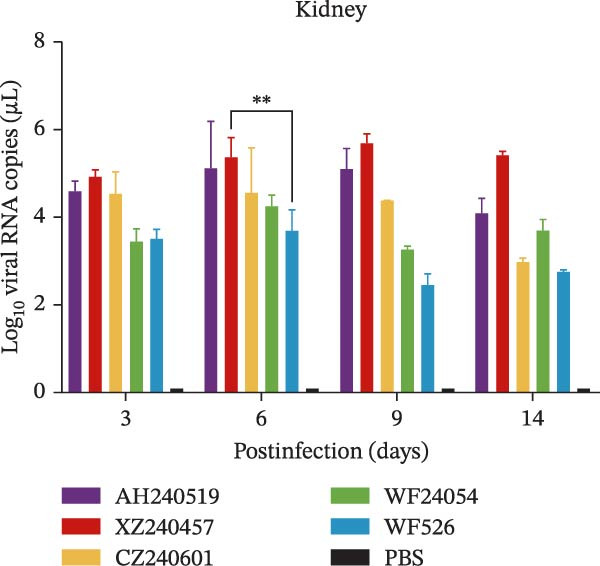
(F)

### 3.6. Pathological Lesions in the Trachea, Lungs, and Kidneys

Autopsy observations at 6 dpi revealed pronounced lesions in all virus‐inoculated groups, except for the WF526‐inoculated group (Figure 5A). At 6 dpi, the tracheas of chickens inoculated with AH240519, XZ240457, CZ240601, and WF24054 exhibited extensive submucosal hemorrhage accompanied by abundant mucoid exudate. In contrast, WF526 induced only a modest level of secretion, indicative of a tempered inflammatory response. Lung pathology was severe across all infected groups, with marked congestion and edema. However, AH240519 and XZ240457 exhibited additional extensive pulmonary necrosis. Kidney lesions manifested as the characteristic “flower‐spotted kidney” phenotype—renal enlargement with multifocal white urate deposition—in all groups except WF526.

Figure 5Gross and histopathological lesions in the trachea, lungs, and kidneys at 6 dpi. (A) Representative gross lesions. The trachea, lung, and kidney tissues from each group are shown; black triangles indicate lesion sites, including tracheal hemorrhage with mucoid exudate, pulmonary congestion and edema, and enlarged kidneys with multifocal urate deposition. No lesions were observed in the PBS group. (B) Histopathology. Tissue sections were stained with hematoxylin and eosin. The trachea: Black arrows indicate mucosal thickening, loss of cilia on the epithelial surface, and infiltration of inflammatory cells. Lung: open triangles denote capillary narrowing, hemorrhage, and infiltration of inflammatory cells. Kidney: Black triangles highlight infiltration of inflammatory cells, necrosis of tubular epithelial cells, and glomerular atrophy.

**Figure:**
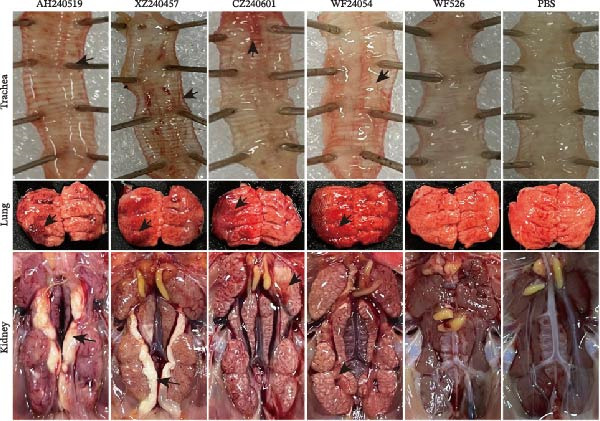
(A)

**Figure:**
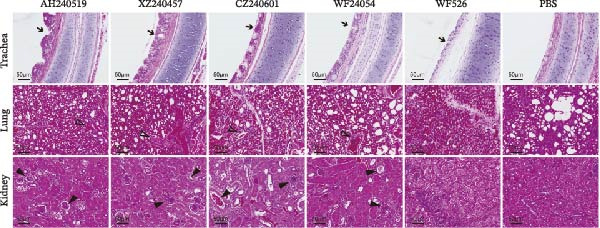
(B)

Histopathological analysis further corroborated these differences (Figure 5B). The tracheal tissues displayed marked mucosal thickening, extensive epithelial desquamation, and dense infiltrates of heterophils and mononuclear cells, indicating profound disruption of the mucociliary barrier. The lung tissues exhibited pronounced capillary engorgement, multifocal hemorrhage, septal thickening, and marked inflammatory cell infiltration, consistent with acute diffuse interstitial pneumonia and compromised respiratory function. The kidney tissues were characterized by glomerular atrophy, widespread tubular epithelial necrosis, and prominent inflammatory infiltrates within the interstitium, underscoring the potential for significant nephron loss and impaired renal excretion. In contrast, tissues from the WF526 group showed only minimal lesions, with largely preserved tissue architecture, minimal inflammatory cell infiltration, and no evidence of extensive epithelial damage or necrosis. Collectively, these observations highlight pronounced virulence heterogeneity among the isolates, with AH240519 and XZ240457 causing the most severe systemic pathology, whereas WF526 displayed minimal pathogenic effects.

### 3.7. Cross‐Neutralization Assay for IBV Isolates and QXL87

The five strains were selected for antiserum preparation to evaluate potential serotype differences. As shown in Table 2, all five strains exhibited cross‐neutralization *R* values exceeding 11% when compared with the QXL87 vaccine strain, indicating that they belonged to the same serotype as QXL87, although the neutralization capacity varied among different IBV strains. The *R* values for CZ240601 and WF526 were 44.75% and 33.98%, respectively, indicating minor subtype differences. In contrast, major subtype differences were observed for AH240519, XZ24057, and WF24054, with *R* values of 13.32%, 23.71%, and 21.50%, respectively, indicating substantial antigenic divergence. The *R* value for AH240519 with QXL87 was the lowest, suggesting that the recombination event allows the virus to escape neutralization by QXL87 through a reduction in S1 gene homology. The S1 amino acid sequence homology between the four GI‐19 IBV isolates and QXL87 ranged from 93.3% to 95.0%, showing a positive correlation with the corresponding *R* values. This correlation suggests that increased S1 amino acid sequence homology is associated with stronger antigenic relatedness. Collectively, these results indicate that QX‐type isolates have evolved into distinct serological subtypes, resulting in reduced antigenic relatedness to the QX‐type vaccine strain.

**Table 2:** Serum cross‐neutralization between IBV isolated strains and vaccine strains.

IBV strain	Serum titer^a^						Antigen relatedness value (*R*) (%) to vaccine strain QXL87^b^
	QXL87	AH240519	XZ240457	CZ240601	WF24054	WF526	
QXL87	2^8.32^	2^5.79^	2^6.67^	2^7.17^	2^6.22^	2^7.00^	100%
AH240519	2^5.22^	2^8.50^	—	—	—	—	13.32%
XZ240457	2^6.00^	—	2^8.50^	—	—	—	23.71%
CZ240601	2^7.00^	—	—	2^8.17^	—	—	44.75%
WF24054	2^6.33^	—	—	—	2^8.67^	—	21.50%
WF526	2^6.78^	—	—	—	—	2^8.58^	33.98%

^a^The titers were determined using reciprocal virus neutralization tests, where serum was diluted and the virus concentration remained constant.

^b^The *R* values were calculated according to the method previously described [24]. The antigenic relationship classification criteria are as follows: >70%, antigenic identity; 33%–70%, minor subtype differences; 11%–32%, major subtype differences; and <11%, no relationship (serotype difference).

### 3.8. Modeling Analysis of the S1 Subunit

A multiple sequence alignment of the S1 amino acid sequences from four QX‐type isolates, one recombinant strain, and the QXL87 vaccine strain revealed distinct amino acid substitutions that underlie the recent antigenic drift (Figure 6A). Eight residues that remained conserved among circulating QX viruses but differed in QXL87 (highlighted by black boxes) were located within the N‐terminal domain (NTD) or C‐terminal domain (CTD) of the S1 subunit. To assess their structural relevance, these substitutions were projected onto a homology‐modeled S1 trimer of QXL87, using the M41 spike cryo‐EM structure (PDB: 6CV0) as a template. Notably, five substitutions—Thr125Ile, Thr143Ser, Leu167Phe, Asp337Glu, and Arg369Lys—were positioned on solvent‐exposed surfaces of the S1 trimer (Figure 6B), suggesting potential roles in receptor recognition and immune escape. Structural modeling of representative GI‐19 S1 proteins further demonstrated that each substitution perturbed the local structure (Figure 6C).

Figure 6Structural modeling analysis of the S1 subunit. (A) ESPript 3.0 alignment of S1 sequences. S1 amino acid sequences of four QX‐type isolates and one recombinant strain were aligned with the vaccine strain QXL87, and eight conserved substitutions present in field isolates are highlighted with black dashed boxes. (B) Surface view of the trimeric QXL87 S1 homologue modeled with SWISS‐MODEL. The subunits were colored in yellow for the S1‐NTD and blue for the S1‐CTD. The five surface‐exposed mutated residues are shown in red. (C) Conformational changes of the five mutations. Close‐up comparisons illustrate the local structural impacts of each mutation. All models were created using SWISS‐MODEL and visualized with PyMOL using PDB 6CV0 as the reference crystal structure mode of IBV S protein.

**Figure:**
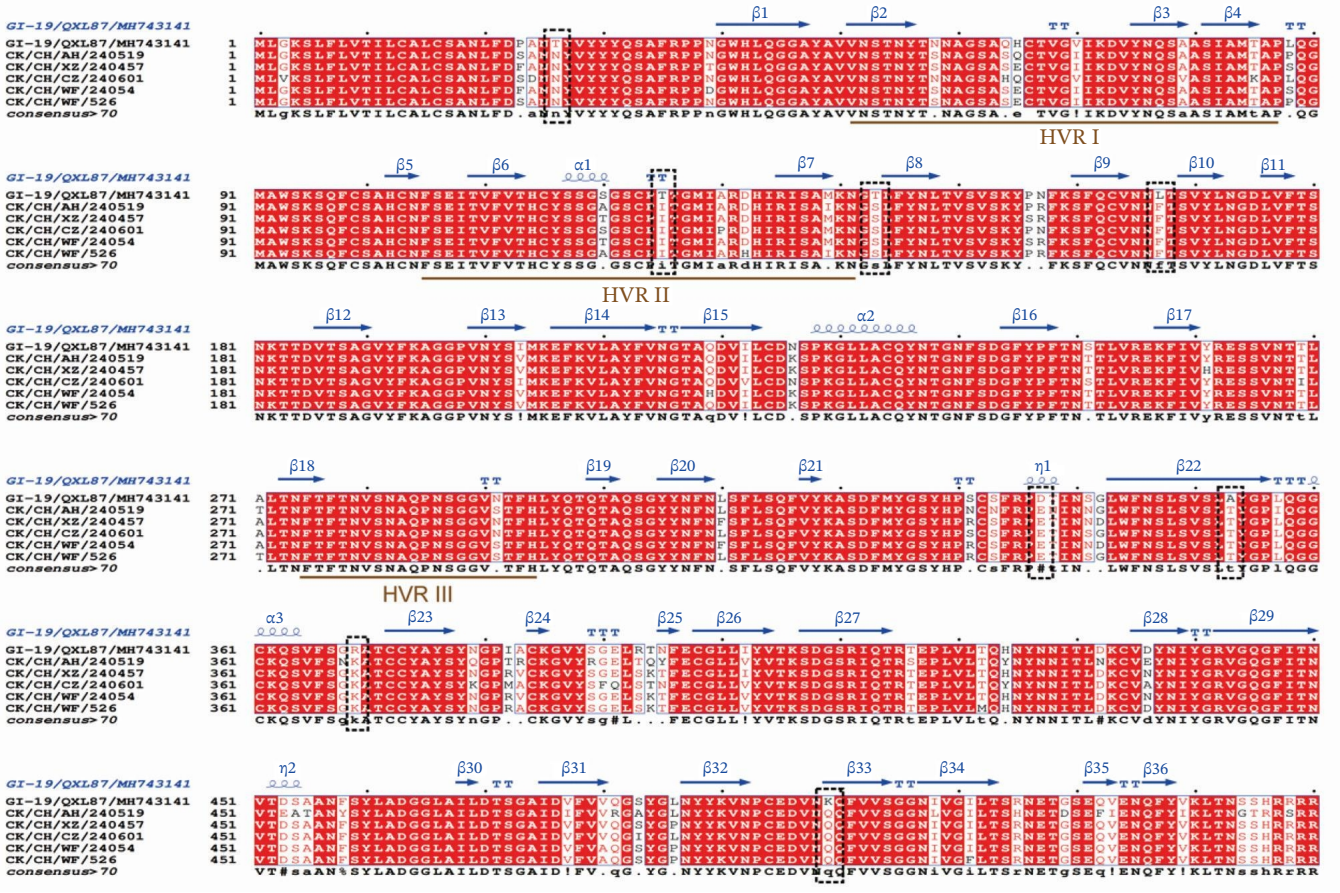
(A)

**Figure:**
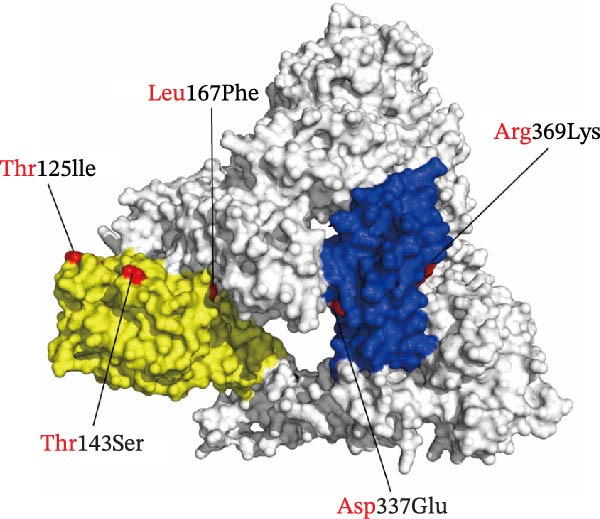
(B)

**Figure:**
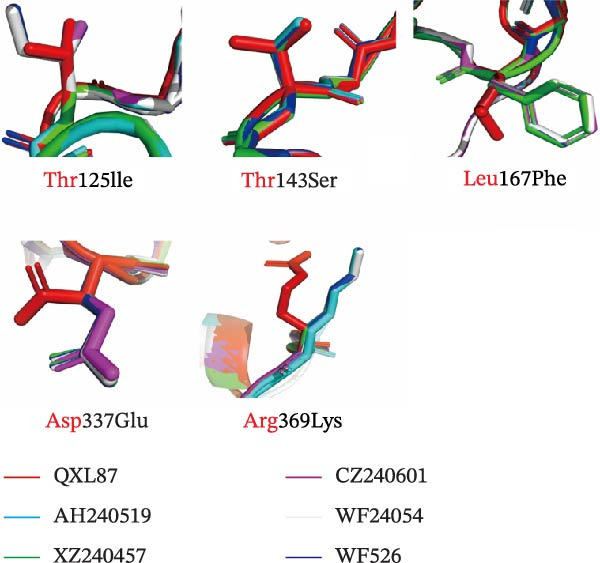
(C)

## 4. Discussion

Since its emergence in China in the 1980s, IB has become one of the most significant diseases threatening the poultry industry [10, 30]. Despite the widespread use of multiple vaccines to control its spread, outbreaks of diverse IB strains continue to be frequently reported [31, 32]. In this study, 49 IBV field strains were isolated from commercial yellow chicken flocks in eastern and southern China between May 2024 and February 2025, despite routine immunization with live‐attenuated H120, 4/91, or QXL87 vaccines. Consistent with the recurrent QX‐type outbreaks in vaccinated flocks, the GI‐13 field isolates showed minimal divergence from the 4/91 vaccine strain, with S1 amino acid identities ranging from 98.9% to 99.1%. In contrast, GI‐19 isolates exhibited significantly lower similarity to QXL87 (93.4%−99.8%), a range that exceeds the 5% threshold generally considered indicative of a serotype shift [14]. This antigenic drift likely underlies the incomplete protection conferred by current vaccines and aligns with reports from other regions of China, suggesting that continual viral evolution remains a major obstacle to long‐term control.

Accordingly, we focused on the genomic and phenotypic characteristics of the QX‐type isolates. Phylogenetic analysis of the S1 gene revealed substantial heterogeneity among 40 QX‐type isolates and the recombinant AH240519, which segregated into five distinct lineages represented by AH240519, XZ240457, CZ240601, WF24054, and WF526. Pairwise S1 amino acid identities to QXL87 were 89.4% (AH240519), 93.9% (XZ240457), 95.0% (CZ240601), 93.3% (WF24054), and 93.9% (WF526), respectively. The steady accumulation of mutations is likely to reshape the antigenic profile of these strains, potentially altering their pathogenic potential. Challenge experiments revealed pronounced virulence heterogeneity among the five phylogenetically distant QX‐type isolates.

Previous studies have indicated that recombination between QXL87 and 4/91 may result in the emergence of highly pathogenic strains [33, 34]. The recombinant strain AH240519 induced 40% mortality, a rate statistically indistinguishable from that of the most virulent parental QX strain. This finding suggests that recombination can preserve or even enhance virulence while simultaneously altering antigenicity [35]. These data reinforce concerns that concurrent use of multiple live vaccines may inadvertently accelerate the generation of novel recombinants.

To elucidate the emergence of such virulent recombinants, it is essential to investigate the underlying molecular mechanisms [8]. Recombination is a vital part of coronavirus replication, crucial for generating subgenomic RNAs (sgRNAs) and being closely associated with the emergence of novel strains. IBV’s error‐prone RNA‐dependent RNA polymerase (RdRp) frequently switches templates during sgRNA synthesis, generating chimeric intermediates and mosaic genomes [15, 36]. The AH240519, WF2407, and CZ240536 strains all resulted from homologous recombination within the S1 gene between the 4/91 and QXL87 vaccine strains. The S1 subunit, which occupies the N‐terminal half of the spike glycoprotein, harbors the major virus‐neutralizing epitopes and constitutes the principal target of vaccine‐induced protective antibodies [37]. However, owing to its role as the predominant target of vaccine‐induced neutralizing antibodies, the S1 subunit is subject to strong immune selection pressure, under which the virus tends to evolve through mutation or recombination to evade immunity conferred by vaccination [38].

The concurrent use of live 4/91 and QX vaccines in China generates a 21–28‐day period during which vaccine virus shedding overlaps with field‐strain co‐infections. The 4/91 S1 domain primarily targets the upper respiratory epithelium [39], whereas the QX S1 domain directs viral replication to renal tubules [8]. A recombination event that fuses these determinants enables progeny viruses to exploit both entry routes, thereby broadening tissue tropism, increasing cumulative viral load, prolonging shedding, and enhancing onward transmission. Unlike point mutants that rapidly attenuate due to fitness costs [40], these S1 recombinants acquire multiple neutralizing epitopes and dual tropism without incremental penalty and can persist endemically within a farm for years [27].

To assess the antigenic relatedness between the circulating isolates and the QXL87 vaccine strain, cross‐neutralization assays were conducted. Although all isolates exhibited *R* values above 11%, indicating the absence of a clear serotype shift, varying degrees of reduced cross‐neutralization activity against QXL87 were observed. Collectively, these antigenic differences among the circulating isolates can be partly attributed to progressive divergence within the S1 subunit. Given the positive correlation between S1 amino acid sequence homology and cross‐neutralization *R* values, such divergence is likely to contribute to the reduced protective efficacy of the QXL87 vaccine against current field strains. However, antigenic analysis of the isolates further indicated that antigenic variation could not be solely determined by the S1 amino acid identity, as certain key residues may exert a pivotal influence on antigenic relatedness [41]. Structural analyses of the five isolates further identified several amino acid substitutions within the S1 subunit. Previous studies have reported that increased heterogeneity within the HVRs is associated with reduced antigenic similarity [42]. In the present study, key substitutions, including Thr125Ile, Thr143Ser, Leu167Phe, Asp337Glu, and Arg369Lys, were located on solvent‐exposed surfaces, inducing local conformational changes that could weaken antibody binding and reduce antigenicity. The Thr125Ile replaced a hydrogen‐bond‐competent threonine with a bulkier hydrophobic isoleucine, distorting the β‐turn backbone [43]. The Thr143Ser mutation caused conformational changes in the β‐sheet structure. The Leu167Phe introduced a rigid phenyl ring that enhanced hydrophobic packing and local rigidity, thereby increasing thermostability but reducing antibody specificity [44]. The Asp337Glu disrupted the local hydrogen‐bond network and destabilized the adjacent η‐helix. The Arg369Lys truncated the side chain, weakening antibody binding and reducing antigenicity [45]. Collectively, these substitutions remodeled key neutralizing epitopes and redefined the antigenic landscape of the S1 subunit. The accumulated divergence has diminished antigenic overlap with the QXL87 vaccine strain, facilitating immune evasion and precipitating the recent QX‐type outbreaks.

The combined evidence indicates that vaccine formulations should be updated to include antigens representative of the dominant clades identified in this study. Ongoing genomic and antigenic surveillance will be essential for detecting antigenic drift and guiding iterative vaccine reformulation.

## Author Contributions

**Zijian Dai**: writing – review and editing, writing – original draft, methodology, investigation, formal analysis, data curation. **Yuanlu Lu**: validation, formal analysis. **Lulu Deng**: validation, formal analysis, conceptualization. **Yan Luo**: resources, investigation. **Yiran Zeng**: investigation, conceptualization. **Yusen Tian, Xianchen Meng, and Haitao Zhang**: visualization, methodology. **Jihui Ping**: writing – review and editing, resources, project administration, funding acquisition, conceptualization.

## Funding

This study was supported by the Xinjiang Uygur Autonomous Region Major Science and Technology Project—Xinjiang Animal Disease Prevention and Control System Quality Improvement Project (Grant 2023A02007), the Breakthroughs in Critical Core Technologies for Jiangsu’s Industrial Development—Development of Multivalent Nanoparticle Vaccines for Major Viral Diseases in Livestock and Poultry (Grant CX(24)1010), and the “Tianchi Talent” Introduction Program.

## Disclosure

Following the use of ChatGPT, the author(s) thoroughly reviewed and edited the content as necessary and take full responsibility for the accuracy and integrity of the publication.

## Ethics Statement

All of the animal experiments were conducted in accordance with the regulations of the administration of affairs concerning experimental animals and approved by the Nanjing Agricultural University Experimental Animal Welfare Ethics Committee with the approval ID: NJAULLSC2025041.

## Conflicts of Interest

The authors declare no conflicts of interest.

## Supporting Information

Additional supporting information can be found online in the Supporting Information section.

## Supporting information


**Supporting Information** Table S1: Primers used in this study. Table S2: IBV reference sequences information. Table S3: Provincial distribution of samples, positivity rates, and genotype‐specific distribution of isolated strains. Table S4: Background and S1 genotypic information of IBV isolates from 2024 to 2025. Table S5: Genetic recombination events of the S1 gene of IBV isolates detected by RDP 4.97 software.

## Data Availability

All data of this study are available from the corresponding author upon request.
