# Supplementary material for: Vaccine‐Associated Recombination and Virulence Heterogeneity of GI‐19 (QX Type) Infectious Bronchitis Virus in Eastern and Southern China, 2024−2025
Source: Transbound Emerg Dis. 2026 Feb 23;2026:8100600. doi: 10.1155/tbed/8100600 (PMC12927963; doi:10.1155/tbed/8100600)
Supplement: Supplementary file 1 — Supporting Information Table S1: Primers used in this study. Table S2: IBV reference sequences information. Table S3: Provincial distribution of samples, positivity rates, and genotype‐specific distribution of isolated strains. Table S4: Background and S1 genotypic information of IBV isolates from 2024 to 2025. Table S5: Genetic recombination events of the S1 gene of IBV isolates detected by RDP 4.97 software. [file TBED-2026-8100600-s001.docx]

**Supplementary Material Tables**

Table S1. Primers used in this study.

| Virus | Primer | Nucleotide sequence (5’-3’) |
| --- | --- | --- |
| AIV | AIV-M-F | AGCAAAAGCAGGTAGATGTTGAAAG |
|  | AIV-M-R | AGTAGAAACAAGGTAGTTTTTTA |
| NDV | NDV-F | GACTTTATGAGCACATCATTCTGGA |
|  | NDV-R | GGGGTTGCACCAGCTGATAGTTGTG |
| IBDV | IBDV-F | ACGAGTTCCTAGCCGAGTGGTCTGA |
|  | IBDV-R | CACCGGTGGCTCTCTTGGCGGGCAG |
| IBV | IBV-S1-F | TTGAARACTGAACAAAAGACMGA |
|  | IBV-S1-R | AATTGATTTGGACCTTATCCATACG |

| Table S2. IBV reference sequences information. | | |
| --- | --- | --- |
| Lineage | Accession numbers (S1) | Strain name |
| GI-1 | FJ888351 | H120 |
|  | GQ504725 | M41 |
| GI-2 | DQ070840 | SDW |
|  | GU393336 | Holte |
| GI-3 | L14069 | GRAY |
|  | L14070 | JMK |
| GI-4 | L18988 | Holte |
|  | AY251816 | GX2-98 |
| GI-5 | MK990809 | Aus/V1/02 |
|  | DQ490215 | V2-02 |
| GI-6 | KF460437 | VicS-v |
|  | DQ515802 | J9 |
| GI-7 | DQ646405 | TW2575/98 |
|  | AY606322 | T07/02 |
| GI-8 | M99484 | SE/17 |
|  | JQ964061 | L165 |
| GI-9 | AY514485 | California/99 |
|  | GQ504721 | Arkansas |
| GI-10 | AF151960 | T6 |
|  | AF151958 | K43 |
| GI-11 | KX258195 | 23/2013 |
|  | JX182783 | UFMG/114 |
| GI-12 | X52084 | D3896 |
|  | MH021175 | D274 |
| GI-13 | KP036504 | CK/CH/LHB/130630 |
|  | KF377577 | 4/91 |
| GI-14 | FN182277 | NGA/324/2006 |
|  | KR231009 | B1648 |
| GI-15 | MN199466 | R18/23 |
|  | FJ807944 | K620/02 |
| GI-16 | AF286302 | Q1 |
|  | GI-16KJ941019 | IZO/28/86 |
| GI-17 | AF027509 | CV-56B |
|  | AF510656 | AL/6609/98 |
| GI-18 | KC577391 | 53XJ-99II |
|  | AY296744 | JP8127 |
| GI-19 | JF732903 | Sczy3 |
|  | AF193423 | QXIBV |
|  | MH743141 | QXL87 |
|  | AY338732 | LX4 |
| GI-20 | AF349620 | Qu16 |
|  | AF349621 | Qu/mv |
| GI-21 | AJ457137 | Italy-02 |
|  | DQ064808 | Spain/98/313 |
| GI-22 | DQ288927 | SAIBK |
|  | GQ265940 | HN08 |
| GI-23 | KY805846 | Ck/EG/CU/4/2014 |
|  | MN509314 | CK/CH/2018/GD03 |
| GI-24 | KF757447 | V13 |
|  | KF809796 | IBV506 |
| GI-25 | KM660636 | GA/10216/2010 |
|  | KP085595 | GA/12274/2012 |
| GI-26 | FN182268 | NGA/BP61/2007 |
|  | FN182270 | NGA/N545/2006 |
| GI-27 | GU301925 | Georgia/08 |
|  | KM660634 | GA/12341/2012 |
| GI-28 | AY702975 | LDT3 |
|  | KX640829 | ck/CH/LGX/111119 |
|  | KR608272 | LDT3-A |
|  | JX291989 | GX-NN-13 |
| GI-29 | KY407558 | I0118/14 |
|  | KC577397 | 66GD-98VI |
| GI-30 | ON470385 | Mex-1 |
|  | ON470386 | Mex-07-1 |
| GII-1 | M21968 | V1397 |
| GII-2 | MK840961 | D181 |
| GIII-1 | U29521 | V18/91 |
|  | U29450 | N1/88 |
| GIV-1 | AF274436 | AR/6386/97 |
|  | U77298 | DE/072/92 |
| GV-1 | DQ059619 | N5/03 |
|  | DQ059618 | N4/02 |
| GVI-1 | GQ265948 | TC07-2 |
|  | KF007209 | SDIB781/2012 |
|  | OL691641 | KrD1515 |
| GVII-1 | MH924835 | I0636/16 |
|  | KM365468 | GX-NN130021 |
| GVIII-1 | ON470392 | Mex-3009 |
|  | ON470391 | Mex-12 |
| GIX-1 | ON470394 | Mex-56-7 |
|  | ON470393 | Mex-14P |

Table S3. Provincial distribution of samples, positivity rates, and genotype-specific distribution of isolated strains.

| Province | Number of samples | Number of farms | Number of positive samples | Positivity rate (%) | Number of isolated strains | Number of genotype-specific strains (n), rate (%) | | |
| --- | --- | --- | --- | --- | --- | --- | --- | --- |
|  |  |  |  |  |  | GI-19 | GI-13 | Recombinant |
| Jiangsu | 160 | 6 | 63 | 39.38% | 12 | n=11, 91.67% | - | n=1, 8.33% |
| Shandong | 128 | 8 | 36 | 28.13% | 13 | n=10, 76.92% | n=2, 15.38% | n=1, 7.69% |
| Anhui | 192 | 12 | 58 | 30.21% | 22 | n=17, 77.27% | n=4, 18.18% | n=1, 4.55% |
| Guangdong | 60 | 2 | 11 | 18.33% | 2 | n=2, 100% | - | - |
| Total | 540 | 28 | 168 | 31.11% | 49 | n=40, 81.63% | n=6, 12.24% | n=3, 6.12% |

Table S4. Background and S1 genotypic information of IBV isolates from 2024 to 2025.

| Isolate | Province | Date | Vaccination history | Clinical type | Type of chicken | Accession no. |
| --- | --- | --- | --- | --- | --- | --- |
| CK/CH/CZ/21 | Jiangsu | 1-May-24 | H120, 4/91, or QXL87 | oropharynx swabs and cloacal swabs | Broiler | PX737632 |
| CK/CH/CZ/507 | Jiangsu | 1-May-24 | H120, 4/91, or QXL87 | oropharynx swabs and cloacal swabs | Broiler | PX737633 |
| CK/CH/WF/464 | Shandong | 1-May-24 | H120, 4/91, or QXL87 | oropharynx swabs and cloacal swabs | Broiler | PX737634 |
| CK/CH/WF/22 | Shandong | 1-May-24 | H120, 4/91, or QXL87 | oropharynx swabs and cloacal swabs | Broiler | PX737635 |
| CK/CH/AH/495 | Anhui | 31-Aug-24 | H120, 4/91, or QXL87 | oropharynx swabs and cloacal swabs | Broiler | PX737636 |
| CK/CH/CZ/0527 | Jiangsu | 31-Aug-24 | H120, 4/91, or QXL87 | oropharynx swabs and cloacal swabs | Broiler | PX737637 |
| CK/CH/AH/596 | Anhui | 31-Aug-24 | H120, 4/91, or QXL87 | oropharynx swabs and cloacal swabs | Broiler | PX737638 |
| CK/CH/CZ/19 | Jiangsu | 31-Aug-24 | H120, 4/91, or QXL87 | oropharynx swabs and cloacal swabs | Broiler | PX737639 |
| CK/CH/AH/23 | Anhui | 31-Aug-24 | H120, 4/91, or QXL87 | oropharynx swabs and cloacal swabs | Broiler | PX737640 |
| CK/CH/AH/532 | Anhui | 20-Oct-24 | H120, 4/91, or QXL87 | oropharynx swabs and cloacal swabs | Broiler | PX737641 |
| CK/CH/AH/29 | Anhui | 20-Oct-24 | H120, 4/91, or QXL87 | oropharynx swabs and cloacal swabs | Broiler | PX737642 |
| CK/CH/AH/520 | Anhui | 20-Oct-24 | H120, 4/91, or QXL87 | oropharynx swabs and cloacal swabs | Broiler | PX737643 |
| CK/CH/AH/48 | Anhui | 20-Oct-24 | H120, 4/91, or QXL87 | oropharynx swabs and cloacal swabs | Broiler | PX737644 |
| CK/CH/WF/755 | Shandong | 20-Oct-24 | H120, 4/91, or QXL87 | oropharynx swabs and cloacal swabs | Broiler | PX737645 |
| CK/CH/CZ/24 | Jiangsu | 20-Oct-24 | H120, 4/91, or QXL87 | oropharynx swabs and cloacal swabs | Broiler | PX737646 |
| CK/CH/AH/754 | Anhui | 20-Oct-24 | H120, 4/91, or QXL87 | oropharynx swabs and cloacal swabs | Broiler | PX737647 |
| CK/CH/AH/769 | Anhui | 20-Oct-24 | H120, 4/91, or QXL87 | oropharynx swabs and cloacal swabs | Broiler | PX737648 |
| CK/CH/AH/768 | Anhui | 20-Oct-24 | H120, 4/91, or QXL87 | oropharynx swabs and cloacal swabs | Broiler | PX737649 |
| CK/CH/WF/614 | Shandong | 15-Dec-24 | H120, 4/91, or QXL87 | oropharynx swabs and cloacal swabs | Broiler | PX737650 |
| CK/CH/CZ/24037 | Jiangsu | 15-Dec-24 | H120, 4/91, or QXL87 | oropharynx swabs and cloacal swabs | Broiler | PX737651 |
| CK/CH/HY/24046 | Guangdong | 15-Dec-24 | H120, 4/91, or QXL87 | oropharynx swabs and cloacal swabs | Broiler | PX737652 |
| CK/CH/AH/603 | Anhui | 15-Dec-24 | H120, 4/91, or QXL87 | oropharynx swabs and cloacal swabs | Broiler | PX737653 |
| CK/CH/AH/518 | Anhui | 15-Dec-24 | H120, 4/91, or QXL87 | oropharynx swabs and cloacal swabs | Broiler | PX737654 |
| CK/CH/WF/526 | Shandong | 15-Dec-24 | H120, 4/91, or QXL87 | oropharynx swabs and cloacal swabs | Broiler | PX737655 |
| CK/CH/CZ/594 | Jiangsu | 15-Dec-24 | H120, 4/91, or QXL87 | oropharynx swabs and cloacal swabs | Broiler | PX737656 |
| CK/CH/AH/0603 | Anhui | 15-Dec-24 | H120, 4/91, or QXL87 | oropharynx swabs and cloacal swabs | Broiler | PX737657 |
| CK/CH/TA/25 | Shandong | 15-Dec-24 | H120, 4/91, or QXL87 | oropharynx swabs and cloacal swabs | Broiler | PX737658 |
| CK/CH/AH/503 | Anhui | 15-Dec-24 | H120, 4/91, or QXL87 | oropharynx swabs and cloacal swabs | Broiler | PX737659 |
| CK/CH/AH/770 | Anhui | 15-Dec-24 | H120, 4/91, or QXL87 | oropharynx swabs and cloacal swabs | Broiler | PX737660 |
| CK/CH/WF/604 | Shandong | 1-Feb-25 | H120, 4/91, or QXL87 | oropharynx swabs and cloacal swabs | Broiler | PX737661 |
| CK/CH/AH/0529 | Anhui | 1-Feb-25 | H120, 4/91, or QXL87 | oropharynx swabs and cloacal swabs | Broiler | PX737662 |
| CK/CH/WF/24054 | Shandong | 1-Feb-25 | H120, 4/91, or QXL87 | oropharynx swabs and cloacal swabs | Broiler | PX737663 |
| CK/CH/WF/240286 | Shandong | 1-Feb-25 | H120, 4/91, or QXL87 | oropharynx swabs and cloacal swabs | Broiler | PX737664 |
| CK/CH/WF/240488 | Shandong | 1-Feb-25 | H120, 4/91, or QXL87 | oropharynx swabs and cloacal swabs | Broiler | PX737665 |
| CK/CH/AH/42 | Anhui | 1-Feb-25 | H120, 4/91, or QXL87 | oropharynx swabs and cloacal swabs | Broiler | PX737666 |
| CK/CH/XZ/240457 | Jiangsu | 1-Feb-25 | H120, 4/91, or QXL87 | oropharynx swabs and cloacal swabs | Broiler | PX737667 |
| CK/CH/CZ/25024 | Jiangsu | 1-Feb-25 | H120, 4/91, or QXL87 | oropharynx swabs and cloacal swabs | Broiler | PX737668 |
| CK/CH/CZ/436 | Jiangsu | 1-Feb-25 | H120, 4/91, or QXL87 | oropharynx swabs and cloacal swabs | Broiler | PX737669 |
| CK/CH/SG/24057 | Guangdong | 1-Feb-25 | H120, 4/91, or QXL87 | oropharynx swabs and cloacal swabs | Broiler | PX737670 |
| CK/CH/CZ/240601 | Jiangsu | 1-Feb-25 | H120, 4/91, or QXL87 | oropharynx swabs and cloacal swabs | Broiler | PX737671 |
| CK/CH/AH/240519 | Anhui | 1-Feb-25 | H120, 4/91, or QXL87 | oropharynx swabs and cloacal swabs | Broiler | PX737672 |
| CK/CH/WF/568 | Shandong | 1-Feb-25 | H120, 4/91, or QXL87 | oropharynx swabs and cloacal swabs | Broiler | PX737673 |
| CK/CH/AH/554 | Anhui | 1-Feb-25 | H120, 4/91, or QXL87 | oropharynx swabs and cloacal swabs | Broiler | PX737674 |
| CK/CH/AH/555 | Anhui | 1-Feb-25 | H120, 4/91, or QXL87 | oropharynx swabs and cloacal swabs | Broiler | PX737675 |
| CK/CH/AH/34 | Anhui | 1-Feb-25 | H120, 4/91, or QXL87 | oropharynx swabs and cloacal swabs | Broiler | PX737676 |
| CK/CH/WF/504 | Shandong | 1-Feb-25 | H120, 4/91, or QXL87 | oropharynx swabs and cloacal swabs | Broiler | PX737677 |
| CK/CH/AH/547 | Anhui | 1-Feb-25 | H120, 4/91, or QXL87 | oropharynx swabs and cloacal swabs | Broiler | PX737678 |
| CK/CH/WF/2407 | Shandong | 1-Feb-25 | H120, 4/91, or QXL87 | oropharynx swabs and cloacal swabs | Broiler | PX737679 |
| CK/CH/CZ/240536 | Jiangsu | 1-Feb-25 | H120, 4/91, or QXL87 | oropharynx swabs and cloacal swabs | Broiler | PX737680 |

Table S5. Genetic recombination events of the S1 gene of IBV isolates detected by RDP 4.97 software.

| Isolated strains | Major parent | Minor parent | Breakpoints (nt) | | Detection methods  (*p* value) |
| --- | --- | --- | --- | --- | --- |
|  |  |  | Beginning | Ending |  |
| AH240519 | QXL87 MH743141 | 4/91 KF377577 | 986 | end | RDP (6.199×10^-37^);  GENECONV (1.217×10^-34^);  BootScan (7.591×10^-04^);  MaxChi (4.105×10^-23^);  Chimacra (3.166×10^-23^);  SiScan (1.232×10^-28^);  3Seq (3.041×10^-57^) |
| WF2407 | 4/91  KF377577 | QXL87  MH743141 | 1 | 123 | RDP (-);  GENECONV (5.590×10^-28^);  BootScan (8.717×10^-29^);  MaxChi (1.008×10^-10^);  Chimaera (1.003×10^-10^);  SiScan (-);  3Seq (1.610×10^-25^) |
| CZ240536 | 4/91  KF377577 | QXL87 MH743141 | 1365 | end | RDP (-);  GENECONV (4.796×10^-32^);  BootScan (-);  MaxChi (8.816×10^-13^);  Chimaera (1.482×10^-12^);  SiScan (7.032×10^-14^);  3Seq (5.525×10^-37^) |
